# Systemic Review of Biodegradable Nanomaterials in Nanomedicine

**DOI:** 10.3390/nano10040656

**Published:** 2020-04-01

**Authors:** Shi Su, Peter M. Kang

**Affiliations:** Cardiovascular Institute, Beth Israel Deaconess Medical Center and Harvard Medical School, 3 Blackfan Circle, CLS 910, Boston, MA 02215, USA; ssu@bidmc.harvard.edu

**Keywords:** biodegradable, nanomaterials, nanomedicine

## Abstract

Background: Nanomedicine is a field of science that uses nanoscale materials for the diagnosis and treatment of human disease. It has emerged as an important aspect of the therapeutics, but at the same time, also raises concerns regarding the safety of the nanomaterials involved. Recent applications of functionalized biodegradable nanomaterials have significantly improved the safety profile of nanomedicine. Objective: Our goal is to evaluate different types of biodegradable nanomaterials that have been functionalized for their biomedical applications. Method: In this review, we used PubMed as our literature source and selected recently published studies on biodegradable nanomaterials and their applications in nanomedicine. Results: We found that biodegradable polymers are commonly functionalized for various purposes. Their property of being naturally degraded under biological conditions allows these biodegradable nanomaterials to be used for many biomedical purposes, including bio-imaging, targeted drug delivery, implantation and tissue engineering. The degradability of these nanoparticles can be utilized to control cargo release, by allowing efficient degradation of the nanomaterials at the target site while maintaining nanoparticle integrity at off-target sites. Conclusion: While each biodegradable nanomaterial has its advantages and disadvantages, with careful design and functionalization, biodegradable nanoparticles hold great future in nanomedicine.

## 1. Introduction

Nanotechnology is being applied in many aspects of human life, including agriculture, transportation, electronics, communication, food industry and medicine [1,2,3,4,5]. Nanotechnology is the manipulation of matter at the nanoscale (1 to 100 nm) to create new particles and devices [5]. Nanotechnology assisted medicine, known as nanomedicine, is an interdisciplinary field of science and technology applying materials at the nanoscale for the diagnosis and treatment of human disease [6,7]. Nanomedicine has emerged as an important aspect of the therapeutic regimen for different types of diseases as it holds great potential for personalized medicine. Nanomedicine also has very diverse applications, including smart imaging, molecular detection, and targeted therapy [7]. Many unique properties of nanoparticles depend on the size and shape, the surface charge and modification, and the hydrophobicity of the nanoparticles [8]. The unique properties of nanoparticles could provide great advantages of nanomedicine. For example, the small size of nanoparticle can allow them cross biological barriers; different structures of nanoparticles can increase the bioavailability of non-soluble or unstable drugs; the modifiable surface of nanoparticles can allow desired targeting capacity to the diseased area for either imaging or specific drug delivery. Improved drug bioactivity, bioavailability and controlled delivery are being realized as drugs can be encapsulated into nanodrug delivery system. It is therefore deemed as a superior therapeutic approach compared to the conventional medicine. With the development of nanomedicine, concerns have also been raised regarding the safety of nanomaterials involved. In the notion of improving the safety profile of nanomedicine, biodegradable nanomaterials are gaining increasing attention in this field. Biodegradable nanomaterials are nano-scale materials that can be naturally degraded under biological conditions in the body [9]. The degradability of the nanoparticles can be a useful property to control cargo release as the ideal biodegradable nanoparticles require efficient degradation at the target site while remaining stable at off-target sites [7,9]. Biodegradable nanoparticles hold great promise in drug delivery system due to a number of reasons: they provide controlled releasing profile; they are stable in the circulation system; they are non-toxic and non-immunogenic; they are also capable of avoiding the reticuloendothelial system, part of the immune system in the body that takes up and clears foreign objects, thus prolonging their circulation time [9]. The rationale for this review is that while there are several reviews on one specific type of biodegradable nanomaterial, there is no recent systemic review on the biodegradable nanomaterials and their applications in nanomedicine [10,11,12,13,14]. In this review, our objective is to evaluate different types of biodegradable nanomaterials that are currently being investigated for their application in different diseases.

## 2. Literature Search Methods

We used PubMed as our source for literature research. The key words used were “biodegradable nanomaterial” and “nanomedicine” and the range of publication date was set within the past 10 years. Out of the 561 studies available at the time of writing, we selected the ones that we considered to be relevant to our review, which reported functionalized biodegradable nanomaterials and their application in nanomedicine. Subsequently, under each subsection, the specific name of the biodegradable nanomaterial were added as a key word to fine-tune the literature search.

## 3. Types of Biodegradable Nanoparticles

Similar to their nondegradable counterparts, biodegradable nanoparticles can be categorized based on their structure and arrangement of the nanomaterials, either by encapsulating the agents of interest as nanocapsule or incorporating those agents into a nanosphere [8]. The agents of interest can either be encapsulated in the nanoparticles or adsorbed on the surface of the nanomaterials [8]. Some examples of classic nanocapsules include micelles and liposomes, and dendrimers are an example of nanospheres. No matter what the structure is or how the payload is being incorporated, biodegradable nanoparticles remain the general advantages of nanoparticles in nanomedicine, such as slow and controlled release of the cargo and targeted delivery, which lead to enhanced therapeutic effects and decreased side-effects, especially for certain cytotoxic drugs, with one more advantage being that biodegradable nanomaterials decrease the cytotoxicity to the body. Further surface modifications can also be done to improve the drug release profile and targeting efficiency.

**Figure 1:**
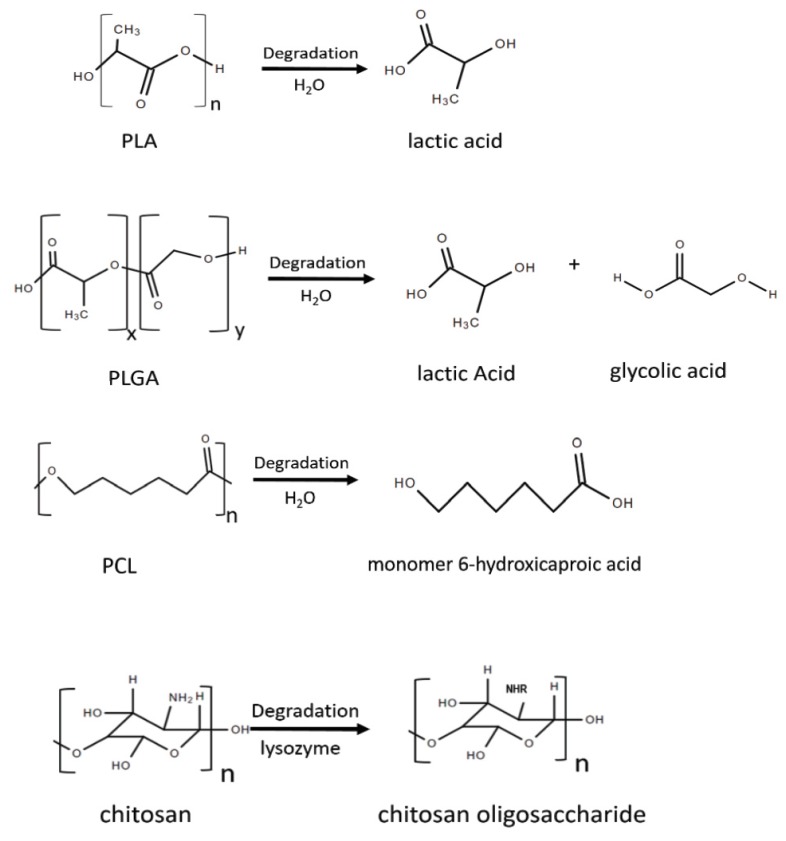
The biodegradation reaction of some commonly used biodegradable nanomaterials.

In making biodegradable nanoparticles, polymers have shown high biocompatibility and biosafety [15]. Polymer-based nanoparticles are solid colloidal particles with a size of 10–500 nm and can be used to carry therapeutic agents of interest by either embedding/encapsulating the agents within their polymeric matrix, or adsorbing/conjugating them onto the surface [15,16]. In addition, the particle surface and size can be modified to control drug release [15]. Based on the main materials used for the formation of the nanoparticles, polymer-based nanomaterials can be categorized into two main groups: synthesized materials such as poly-D-L-lactide-co-glycolide (PLGA), polyactic acid (PLA), and poly-e-caprolactone (PCL); and natural materials like chitosan. All these polymers can undergo degradation process to be degraded into products that can be safely processed in the body (Figure 1). The degradation rate of polymer-based nanoparticles are affected by many factors, including internal factors such as the size, structure and molecular weight of the nanoparticles, as well as external factors, such as pH and temperature, both of which influence the payload releasing profile [17]. Synthetic polymers have the general advantage of relatively long drug release period, compared to their natural polymer counterparts [18]. However, based on the type of materials being applied, certain disadvantages may also arise for certain nanoparticles, whether it is low drug-loading capacity, instability, or increased fragility. The existence of different advantages and disadvantages of different nanomaterials require careful consideration in order to choose proper nanomaterials when designing new nanoparticles. The detailed advantages and disadvantages of each nanomaterial will be summarized in the following sections (Table 1).

### 3.1. Micelles

**Figure 2:**
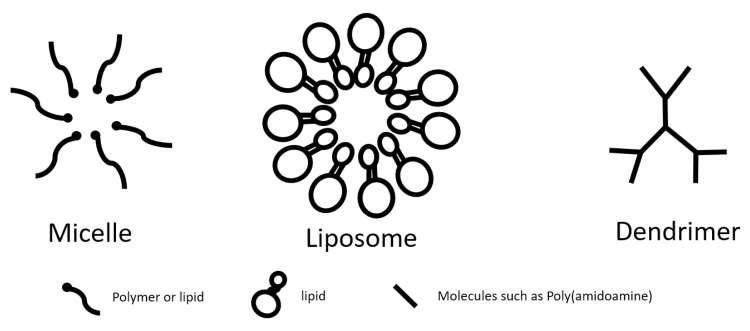
Examples of applications of nanomaterials in different nanoparticles.

A micelle is defined as a collection of amphiphilic molecules that can self-assemble in water into a spherical vesicle [19]. Micelles can be formed by either lipid- or polymer-based amphiphilic molecules [20,21,22]. Lipid-based micelles are composed of small molecules that have a hydrophilic head group and a hydrophobic tail, which is the hydrocarbon portion of long fatty acids [23]. Polymer-based micelles are formed with polymers of alternating hydrophilic, such as poly(ethylene oxide) (PEO), and hydrophobic blocks, such as poly(propylene oxide) (PPO), poly(lactic acid) (PLA), or other biocompatible and hydrophobic polyethers or polyesters [23] (Figure 2). Polymeric micelles range from 10 to 100nm in size and have a narrow size-distribution [17,24]. Self-assembly of the single chains of amphiphilic molecules occurs when they reach certain concentrations, which are defined as the critical micelle concentration (CMC) [17]. CMC is an important parameter to assess the stability of micelles as micelles with lower CMC are more thermodynamically stable [17]. The molecules of micelles are self-assembled in a way that the core is hydrophobic whereas the shell is hydrophilic [17]. The hydrophobic core allows encapsulation of poorly soluble drugs whereas the hydrophilic shell increases circulation time and structural stability to enable controlled and sustained release of the drugs, though their circulation time is shorter than that of liposomes due to their smaller size [20,22].

#### 3.1.1. Polylactic Acid (PLA) Micelles

Poly(D,L-lactic acid) (PLA) is a type of biodegradable nanomaterial that is widely used in nanomedicine. PLA is produced from the monomer of lactic acid (LA), which is obtained from glucose fermentation [25]. In the process of synthesizing PLA, LA is converted to lactide and eventually to PLA [25]. Under physiological conditions, PLA can be hydrolyzed into lactic acid, and eventually secreted out of the body [26,27].

PLA is relatively hydrophobic, and is therefore commonly used for implants (such as stents or screws for bone fixations), medical sutures, as well as drug delivery micelles as it improves oral bioavailability of hydrophobic drugs [10,11]. One of the advantages of using PLA to make micelles is that its physical and chemical properties, such as size, shape, molecular weight and liquid-to-gas ratio, can all be easily altered to obtain desirable pharmacokinetic and biodegradable properties [11]. However, limitations also exist for PLA nanoparticles, such as non-specific uptake by the reticuloendothelial systems, as well as low drug loading capacity and low encapsulation efficiency [11]. Although PLA generally elicits low immunotoxicity, it has been shown that the size of PLA nanoparticles affects their immunotoxicity- the smaller the nanoparticles, the more immunotoxic they are [28].

As PLA degrades primarily by hydrolysis while the polymer degradation rate is determined by its reactivity with water, any factors that can change the reactivity can affect the degradation rate [10]. Although the release of the drug in micelles is mainly controlled by the rate of diffusion of the drug from the micellar core and the rate of biodegradation of the micelles, other factors such as the compatibility between the drug and core forming block of copolymer, the amount of drug loaded, the molecular volume of the drug, and the length of the core forming block also affect the drug release profile [29]. A general consideration when choosing PLA as micelle block is to match the mechanical properties and the degradation rate to the need of the application [10]. Meanwhile modifications of the nanoparticles have also been made for better delivery efficiency. For example, micelle-templated Polylactic-co-glycolic acid (PLGA) nanoparticles have been developed for hydrophobic drug delivery with increased stability and loading capacity [21].

#### 3.1.2. Polylactic-Co-Glycolic Acid (PLGA) Micelles

Polylactic-co-glycolic acid (PLGA) is one of the best characterized biodegradable polymers that is frequently used for drug delivery as it can be hydrolyzed in the body to produce metabolite monomers lactic acid and glycolic acid, and eventually degraded into non-toxic products (i.e., water and carbon dioxide) that can easily be eliminated from the body [8,30,31]. PLGA is a copolymer of hydrophobic polylactic acid (PLA) and hydrophilic polyglycolic acid (PGA) [32]. PGA is also a biodegradable material that can be degraded into glycolic acid, which is a natural metabolite [33]. For this reason, PGA has been most commonly used in the production of resorbable sutures [33].

As a copolymer, PLGA has a wide range of erosion time and modifiable mechanical properties [31,32]. The degradation rate of PLGA can be changed by adjusting the ratio of PLA:PGA and their molecular weights in order to control the release of incorporated drugs [31,32]. These characteristics make PLGA a very attractive type of material for drug delivery [31,32]. Different approaches of loading systems exist for PLGA-based nanoparticle therapeutics, including protein encapsulation, protein adsorption, and nucleic acid loading [12,34].

Despite the fact that more than three decades have passed since PLGA first received the approval from the Food and Drug Administration (FDA), there are still only 19 long releasing approved products containing PLGA [35,36]. The slow development of long releasing PLGA drug carriers are attributed to the challenge that regardless of its many modifiable properties, the acidic PLGA monomers are not suitable for certain drugs and bioactive molecules [8]. Another major challenge of formulating PLGA containing drugs still lies in the difficulty of achieving the desired drug release profile [36]. The biodistribution and pharmacokinetics of PLGA are non-linear, dose-dependent, and easily affected by different factors including the hydrophilicity, the inter-hydrolytic group chemical interactions, the crystallinity as well as the volume to surface ratio of PLGA [31].

#### 3.1.3. Modification of Micelles

Poly-ethylene-glycol (PEG) is commonly used to modify the surface of nanoparticles to enable long-term circulation [8]. The process of incorporating PEG onto the surface of a nanoparticle is known as PEGylation [8]. PEGylation has been incorporated for the development of various block copolymers [31]. PEG-b-PLA micelles are used as a platform for the systemic multi-drug delivery of poorly water soluble anticancer agents because PEGylation stabilizes micelles and improves encapsulation capacity [37]. PEG-PLGA copolymers can form nanospheres, micelles and hydrogels, making them great biodegradable nanomaterials for the construction of nanodrug delivery system [38]. Depending on the structural differences, PEG-PLGA copolymers can have characteristics suitable for different loading agents, enabling multi-drug loading capacity, further improving their therapeutic efficacy [38]. For examples, PLGA-b-PEG-b-PLGA is a thermosensitive copolymer that can transition from solution into gel at body temperature for multi-drug delivery of both hydrophobic and hydrophilic anticancer agents [37]. Several such PEG-PLGA copolymer composed multi-drug delivery systems have been approved by the FDA as neoadjuvant therapy for cancer treatment [37].

In some micelles, functionalized PEG layers are added as the hydrophilic outer shell to attain receptor-mediated drug and gene delivery through PEG-conjugated ligands with a minimal non-specific interaction with other proteins [39]. Moreover, in order to meet the needs of delivering different types of drugs, other types of micelles have also been developed [40]. For example, in addition to conventional micelles with a hydrophobic core and a hydrophilic shell, there are also reverse micelles with a hydrophilic core and a hydrophobic shell to ensure sustained drug release through the hydrogen bond between the drug and the core [40]. In addition, since tumors have lower pH compared to healthy tissues, micelles can also be functionalized by adding peptides responsive to pH change for effective cancer imaging and therapy [41]. All these modifications enable pre-clinical evaluation and clinical translation of emerging agents [37].

### 3.2. Poly-ε-Caprolactone (PCL) Nanoparticles

Poly-ε-caprolactone (PCL) is a polymer member of the aliphatic polyester family that is typically obtained by polymerization processes using a monomer and an initiator [13]. PCL can also be biodegraded, by hydrolysis of its ester linkage, into 6-hydroxycaprioc acid and then into acetyl-CoA, which eventually becomes water and carbon dioxide via the citric acid cycle [42,43]. Particularly, unlike PLA and PLGA, the degradation of which produce acidic products that further catalyze the polymer degradation process, the degradation of PCL does not produce acidic byproducts, making PCL a more favorable nanomaterial for the development of long-term implantable devices for its slow degradation rate [8,17]. Cholic acid can functionalize branched PCL with different molecular weights to meet the need of different nanodrug delivery systems, as higher molecular weight of the polymer matrix results in a slower drug release rate [44].

Since PEGylation of nanoparticles can be used to reduce immunogenicity and toxicity, prolong circulation time, change bio-distribution and optimize nanoparticle activities, copolymers of hydrophilic PEG and hydrophobic PCL can yield high biocompatibility and biodegradability [45]. The high biocompatibility, biodegradability, long circulation time and easy modification of surface properties of micelles composed of PEG-PCL di-block copolymers make them favorable candidates as nanodrug delivery systems [45].

### 3.3. Chitosan Nanoparticles

Derived from natural biopolymer chitin, chitosan is a copolymer of D-glucosamine and N-acetylglucosamine bonded via the β(1–4) linkages [16,46]. It can be degraded in vivo by several enzymes, mainly by lysozyme, a protease that ubiquitously exists in mammalian tissues [46]. Lysozyme hydrolyses the β(1–4) linkages between N-acetylglucosamine and glucosamine in chitosan to produce oligosaccharides, which can then either be excreted or be part of glycosaminoglycans or glycoproteins [46,47].

Chitosan has good absorbability, permeability, moisture retention and are easily degradable [15]. It shows low toxicity in both in vitro and in vivo models [14]. However, it is very sensitive to environmental temperature and is recommended to be stored at low temperatures [47]. Generally, the drug delivery system of chitosan nanoparticles is similar to the PLGA system, but the chitosan system is more pH dependent [14]. Chitosan nanoparticles have been applied in many site specific drug delivery systems via different administrative routes, including oral, nasal, and pulmonary drug delivery systems [14]. The mucoadhesive properties of chitosan nanoparticles increase the absorption rate of the drugs in the intestine [14].

PEG can be incorporated to increase the stability of the chitosan nanoparticles [46]. Chitosan oligosaccharide can also be functionalized to be a “switch on” imaging agent by conjugating with aggregation induced emission active tetraphenylethene (TPE) and lipophilic–cationic triphenylphosphonium (TPP) molecules [48]. Once chitosan self-assembles, TPE provides self-assembly induced fluorescence and TPP helps the nanoparticle enter into the cell by lipid-raft endocytosis [48].

Since the environmental pH affects the degradation rate of chitosan, considerations need to be made when designing chitosan nanoparticles for their encapsulated drugs to be effectively released at the diseased site, especially when the microenvironment of the drug releasing site is acidic, for example, the acidic tumor site [46]. Other pH variables can also limit the development of chitosan nanoparticles. For example, the orientation of β(1–4) linkages of chitosan can change under physiological pH depending on the crosslinkers used in the formation of chitosan nanoparticles [46]. The change in orientation can decrease the accessibility of the β(1–4) linkages to lysozyme, which can then lead to limited degradation of nanoparticles, since the breaking of the β(1–4) linkages by lysozyme is essential to the degradation of chitosan nanoparticles (Figure 1) [46]. In addition, poor long-term stability is a major drawback for large manufacture of chitosan nanoparticles [46]. Therefore, further investigations are still needed to fully understand the mechanisms of the interactions between chitosan and lysozyme when designing chitosan nanoparticles.

### 3.4. Dendrimers

Dendrimers are the smallest of nanocarriers that present as sphere-shaped and are structurally similar to branching polymer chains [49]. They are between 1 nm to 100 nm of diameter in size [50]. These radially symmetric molecules with well-defined, homogeneous, and monodisperse structure consisting of tree-like branches are analogous to protein, enzymes, and viruses, and can be easily functionalized [51]. Because their amphiphilic copolymers have both hydrophilic and hydrophobic monomer units, they can be used to carry drugs with poor solubility [49]. Drugs can be incorporated into dendrimers by covalent binding, electrostatic interactions or encapsulation [50]. The branch structure gives dendrimers a very high surface-to-volume ratio, enabling them to increase drug carrying efficiency [49]. In addition, their high degree of branching, polyvalency, biocompatibility, high water solubility, and low immunogenicity make dendrimers excellent vehicles for safely and effectively transporting drugs, and they are particularly attractive as MRI imaging agents [50,52].

Dendrimers are transported into and across cells via endocytic pathways [52]. Depending on the charge and modifications made on the surface of dendrimers, cytotoxicity may occur during the interactions with cell membrane [50]. Poly(amidoamine) (PAMAM) dendrimers are the most commonly used dendrimer in nanomedicine [50]. PAMAM has been applied in many drug or gene delivery systems and can be administered through various routes [50]. It can also be applied in the treatment of inflammatory diseases such as atherosclerosis and rheumatoid arthritis because its structure exerts anti-inflammatory activity [50]. Furthermore, arginine functionalized peptide dendrimers can condense plasmid DNA and protect it from nuclease digestion, which can serve as potential gene delivery vehicles [53].

### 3.5. Lipid-Based Nanoparticles

Besides polymer-based nanoparticles, lipid-based nanoparticles such as liposomes have also been employed in the drug delivery system for decades. As lipid is generally considered safe in the human body, for example, lipoproteins are natural nanoparticles that are found inside the human body, lipid-based nanoparticles have been under development as drug delivery systems [24]. Liposomes are spherical vesicles ranging from 10 to 1000 nm that consist of one or more phospholipid bilayers [24]. The phospholipid can be naturally occurring or synthetic phospholipids such as phosphatidylcholine (PC), phosphatidylethanolamine (PE), phosphatidylserine, and phosphatidylglycerol [54]. Cholesterol is generally added to stabilize the lipid bilayers of liposomes [54]. The aqueous core of liposomes can load and hold hydrophilic agents while its lipid bilayers can load hydrophobic agents [55].

Due to the similarities between the composition of liposome and that of cell membranes, liposomes are considered more biocompatible than other synthetic materials [55]. They are non-hemolytic, non-toxic, non-immunogenic, biocompatible and biodegradable [49]. Liposomes can self-assemble, which enables easy drug loading. They can carry large drug payloads and protect their encapsulated drugs from early inactivation, degradation and dilution in the circulation and can be formulated into different forms for different routes of administration [49,56]. Liposomes can be applied in a wide range of areas. They can not only be utilized to carry low molecular weight drugs, imaging agents, peptides or nucleic acids, but also serve as part of surgical implants for tissue repair, or as biosensors [56,57,58,59].

In addition to PEGylation and size alteration, liposomes can also be functionalized by attaching certain chemistry functional groups, peptides, antibodies, or acids to their surface to improve cell targeting efficiency [55,60]. Similar to the antigen-antibody complex formed between the antibody conjugated to the nanoparticles and the antigen presented on the surface of the cell, chemistry functional groups conjugated to the surface of the nanoparticles can form strong covalent bonds with metabolically labelled cell surface glycans [60]. Such reaction is known as “click” chemistry [60]. Compared with antigen-antibody complex, the “click” chemistry requires fewer functional groups, and form stronger bonds to allow sufficient time for the encapsulated nano-drugs to be internalized into the cells [60]. Recently, Boyd’s groups functionalized liposomes containing drug nanocrystals using PEGylation and attachment of azide functional groups to improve drug loading capacity and to achieve cell-targeted delivery [60].

However, while liposomes have high loading efficiency, their low stability, fast clearance rate and a complex method of fabrication limit their potential in industrial scale fabrication [24]. To overcome the challenges faced by liposomes, solid lipid nanoparticles (SLNs) and nanostructured lipid carriers (NLCs) have subsequently been developed [24]. SLNs and NLCs have higher stability and lower toxicity compared to polymeric nanoparticles due to their smaller size and natural materials [24]. However, their low encapsulation efficiency hinders their potential to be widely pursued in the biomedical field [61]. Since both lipid-based and polymer-based nanoparticles have their own limitations, lipid-polymer hybrid nanoparticles have recently been developed to provide wider opportunities for the biomedical applications [62]. New polymer liposomes such as electrostatically crosslinked polymer–liposomes have also been developed [63]. These pH-sensitive copolymer methoxy poly(ethylene glycol)-block-poly(methacrylic acid)-cholesterol (mPEG-b-P(MAAc)-chol) and crosslinking reagent poly(ethylene glycol) end-capped with lysine (PEG-Lys2) were crosslinked into polymer–liposomes through electrostatic interactions [63]. These polymer-liposomes are stable under physiological conditions, but breaks down under acidic condition- similar to tumor microenvironment- to rapidly release their payloads, offering a new approach for anti-cancer therapies [63].

### 3.6. Other Natural Materials

Similarly, other natural biodegradable materials such as gelatin are also widely used in the nanomedicine field. Gelatin is a denatured protein that can be obtained either by partial acid or alkaline hydrolysis or by thermal or enzymatic degradation of animal collagen protein [64]. Derived from collagen, the most abundant protein in animals, gelatin does not produce any harmful by-products upon enzymatic degradation in the body [64]. Gelatin is considered as GRAS (generally regarded as safe) by the FDA [65]. Because gelatin is stable, easily modifiable and biodegradable, it is involved in the development of many clinical applications, including drug delivery system and hydrogels [64]. Right now the challenge that limits gelatin nanomedicine production is to make commercial gelatin nanoparticles homogenous in size [64].

## 4. Applications of Biodegradable Nanoparticles

Depending on the size, shape and composition/structure of the nanoparticles, different nanoparticles have different encapsulation efficiency, pharmacokinetics, and releasing mechanism. For example, the administration and encapsulation efficiency, as well as stability are different for nanoparticles that are composed of PGLA and those of PCL [8]. With the add-on targeting properties of these biodegradable nanoparticles, biomedical applications such as imaging as well as targeted drug delivery have been greatly advanced. However, in the clinical application of the biodegradable nanoparticles, while the safety profile of these nanoparticles has improved due to the biodegradability of the nanomaterials used, for the very reason, general challenges persist regarding the circulation time as well as the drug incorporating and/or releasing efficiency as they need to compete with the degradation rate of the nanomaterials.

### 4.1. Imaging

One of the early applications of nanomedicine involves the use of nanomaterials as contrast agents in biomedical imaging for diagnosis purpose, as the easy modification and targeting property of nanoparticles enable them to localize the tissue of interest and visualize with high resolution [7]. These nanoparticles are applied in many imaging modalities such as computed tomography (CT), magnetic resonance imaging (MRI), positron emission tomography, fluorescence imaging and photoacoustic imaging [7]. Targeted delivery by nanocarriers can greatly reduce the concentration of contrast agents, reducing the risk of contrast-induced kidney injury [66]. Gold nanoparticles are central in the development of imaging contrast agents [7]. However, if the nanoparticle itself cannot be biodegraded, long-term safety concern still remains. For instance, although metal nanoparticles with surface plasmon resonance in the near-infrared region (NIR) were of great interest for imaging, not being biodegradable raised concerns for the long-term safety of these nano-agents [67]. In 2010, a platform was first developed to synthesize metal/polymer biodegradable nanoclusters smaller than 100 nm with strong NIR absorbance for multimodal application [67]. With the safety issue gaining greater attention in the development of nano-imaging contrast agents, imaging agents underwent further development. Soon after that, biodegradable polydisulfide dendrimer nanoclusters were developed as MRI contrast agents to overcome safety concerns related to nephrogenic systemic fibrosis [68].

Photoacoustic imaging has also emerged as a promising imaging platform with a high tissue penetration depth [69,70]. It applies both NIR laser and ultrasound for the imaging purpose. The use of polymers made it possible to develop tunable and biodegradable gold nanoparticles as contrast agents for both CT and photoacoustic imaging [71]. Recently, not only polymers, but also natural materials are being engineered as biodegradable imaging agents. Fathi et al. have recently developed a photoacoustic imaging nanoprobe from nanoprecipitation of biliverdin, a naturally occurring heme-based pigment, which can be completely biodegraded to biliverdin reductase, a ubiquitous enzyme found in the body [69]. Excitation at near-infrared wavelengths leads to a strong photoacoustic signal, while excitation with ultraviolet wavelengths results in fluorescence emission [69]. In vivo experiments demonstrated that these nanoparticles accumulate in lymph nodes, suggesting that they can be used as a means to detect metastasized cancer [69]. Similarly, MTP1, a tumor metastasis targeting peptide, has been employed to modify the indocyanine green (ICG)-loaded PEG-PLGA micelles for targeted imaging of cervical cancer and metastasis [72].

One major factor that limits the efficacy of particle-based agents is their rapid sequestration by the mononuclear phagocytic system [73]. Even though low-fouling polymers such as PEG can reduce the immune recognition and clearance, these nondegradable polymers can accumulate in the human body and may cause adverse effects after prolonged use [73]. To overcome this challenge, Bonnard et al. used a recombined protein with the amino acid repeat proline, alanine, and serine (PAS) cross-linked into particles with lysine (K) and polyglutamic acid (E) [73]. The obtained PASKE particles have a prolonged circulation time and can be rapidly degraded in the cell’s lysosomal compartment [73]. When combined with near-infrared fluorescent molecules and an anti-glycoprotein IIb/IIIa single-chain antibody targeting activated platelets, the PASKE nanoparticles was able to image carotid artery thrombosis in a mouse model, demonstrating its potential as a promising biodegradable tool for molecular imaging of vascular diseases [73].

Not only in cancer field, nano-technology assisted imaging has also been applied in atherosclerosis and other cardiovascular disease [74,75]. With the help of a tumor homing peptide, micelles have been shown effective in targeting not only the tumor site, but also at the plaques of atherosclerosis. The targeting property is realized by adding a peptide that homes to plaques- a clot-binding peptide cysteine-arginine-glutamic acid-lysine-alanine (CREKA) [76]. When CREKA is directly bound to the MRI contrast agent, it has been shown to be effective in detecting breast tumor [77]. Depending on the loading agents, micelles can help diagnose atherosclerosis if loaded with dyes, and decrease the plaque size if loaded with drugs [76]. This peptide was identified as a tumor-homing peptide by in vivo phage library screening, and subsequently it was shown to bind to clotted plasma proteins in the blood vessels and stroma of tumors [76].

### 4.2. Theranostics

A new concept- theranostics- the ability of “see and treat”- has become a well sought-after model in developing new multifunctional nanomedicine. These smart nanoparticles combine imaging agents, payload drugs and targeting moieties to accomplish diagnosis together with therapy delivery [7]. They can be engineered to be triggered in response to environmental changes such as pH, temperature, light, and ultrasound [7]. Some smart PLGA-based nanoparticles have been developed, including PH-responsive, thermos-sensitive and light-responsive nanoparticles [78]. In cancer treatment, many biodegradable polyacrylamide nanocarriers are applied for theranostics [79,80,81]. Nanovesicles are being developed for photoacoustic imaging and photothermal therapy (PTT), a therapeutic method that induces cell death using the heat energy converted from absorbed light energy, to enable minimum invasive cancer therapy [82]. The disulfide bond at the terminus of PEG-b-PCL copolymer can allow dense packing of gold nanoparticles, therefore enabling simultaneous photoacoustic imaging as well as enhanced PTT [82]. The designs of the nanoparticles have also been continuously improved to enhance biodegradability and efficacy of PTT [83]. Using biodegradable photonic melanoidin nanoparticles, Lee et al. were able to image lymph nodes and GI track, and to perform tumor ablation and photothermal lipolysis [84]. Recently, biomimetic mineralization method has also been applied to develop biodegradable multifunctional anti-tumor nanoparticles. Using this concept, Fu et al. developed a biodegradable manganese-doped calcium phosphate nanoparticle that can be used both as an MRI contract agent and an anti-tumor drug [85].

Applying 3D printing technology, Ceylan et al. designed a gelatin hydrogel-based, magnetically powered and controlled microswimmer, responsive to the pathological markers in its microenvironment for theranostic cargo delivery in cancer diagnosis and treatment [86]. This microswimmer can be biodegraded by matrix metalloproteinase-2 (MMP-2) enzyme, an enzyme that is highly expressed in breast cancer [86]. At normal physiological concentrations, MMP-2 can degrade the microswimmer to soluble nontoxic molecules [86]. If the MMP-2 concentration reaches pathological level, the microswimmer rapidly responds by swelling and thereby boosting the release of the embedded cargo molecules [86]. Banik et al. recently reported a multifunctional dual-targeted HDL-mimicking PLGA nanocomplex with both mitochondria and macrophage-targeting surface functionalities loaded with MRI contrast agent to achieve target-specific MRI contrast enhancement as well as lipid removal property for the treatment of atherosclerosis [87].

### 4.3. Targeted Delivery System

Nanomedicine using biodegradable polymeric nanoparticles as drug delivery systems have been engineered to treat cancer via multiple approaches: to target cancer cells, or the blood vessels that supply the nutrients and oxygen that support tumor growth, or immune cells to promote anti-cancer immunotherapy [88]. The use of biodegradable nanoparticles for targeted anti-cancer therapies yielded some clinical trials [88]. The encapsulation approach using PLGA can help prolong the circulating time of drugs that are unstable under the physiological condition and to minimize the side effects of certain drugs [30]. For example, 9-Nitrocamptothecin (9-NC) is a family of anticancer agents with low stability at biological pH and low water solubility [89]. PLGA encapsulation improves the drug release profile of 9-NC up to 160 h [89].

Similarly, in the field of cardiovascular disease, targeted nanodrug delivery system is under active investigation. To minimize the adverse effects while maximizing the drug effects, nanoparticles could to be superior as drug delivery systems compared to conventional drugs. For the treatment of cardiovascular disease (CVDs), current goals are focused on restoring normal blood flow to the heart as well as the prevention of recurrent cardiovascular insults [20]. Antithrombotic therapy is the first-line treatments for the prevention of CVDs, but they also significantly increase the risk of bleeding [20]. It remains a great challenge to effectively balance the ischemic risk reduction and the risk of bleeding [90]. Situations like these call for the need of developing nanomedicine that can target the disease area for drug delivery yet minimizing the side effects. Several drugs are delivered via the liposome drug delivery system for the treatment of angina pectoris. Takahama et al. encapsulated amiodarone, an anti-arrhythmic drug, in conventional liposomes to treat rat models that had undergone cardiac ischemic/reperfusion procedure, and showed reduced morality rate in the treated group that was due to lethal arrhythmia and the negative hemodynamic changes- the common side effects of amiodarone [91]. PLA has also been applied to encapsulate the drug for restenosis [49]. Several anti-inflammatory nanomedicines have been developed for targeted treatment of atherosclerosis, ischemia/reperfusion and post myocardial infarction left ventricular remodeling [32]. However, so far the targeted therapy in cardiovascular diseases using nanomaterial-based drug delivery vehicles have only shown effectiveness in preclinical settings [92]. Limitations lie in the gap in the knowledge of clinical safety, the requirement of composition purity and long-term stability of payload, as well as challenges and cost in scaled up production [93]. Recently, by scaling up the animal models from murine to rabbit and porcine, Muldler’s group has taken the imaging-assisted nanotherapy one step closer to be realized in clinical settings [94].

#### 4.3.1. Antioxidant Delivery

Oxidative stress has been associated with cytotoxic effects of cellular exposure to engineered nanomaterials [95]. After entering human body, changes in structural and physicochemical properties of nanoparticles can lead to changes in biological activities including the generation of reactive oxygen species (ROS) [96]. In this respect, if the nano-drug delivery system can deliver agents that combat oxidative stress, it can alleviate cell injury induced by excessive ROS. Kang’s group has developed a new type of nanoparticle, named PVAX, which was formulated from copolyoxalate containing vanillyl alcohol (VA), an antioxidant extracted from natural herbs [97,98]. VA and the H_2_O_2_-responsive peroxalate ester linkages are incorporated covalently in the backbone of PVAX [97,98]. When encountered high levels of H_2_O_2_ at the sites of ischemia/reperfusion (I/R), PVAX is degraded and releases VA, exerting anti-inflammatory and anti-apoptotic activities [97,98]. PVAX has shown effectiveness in different types of I/R injuries, including hind limb I/R, liver I/R as well as cardiac I/R [97,98]. Andrabi et al. used biodegradable nanoparticles (nano-SOD/CAT) to encapsulate the antioxidant enzymes superoxide dismutase (SOD) and catalase (CAT) to effectively deliver those enzymes at the lesion site to protect mitochondria from oxidative stress, therefore protecting the spinal cord from secondary injury [99]. Tapeinos et al. have developed biodegradable PLGA microspheres coated with collagen type I and MnO_2_ nanoparticles to scavenge ROS and protect cells from apoptosis induced by oxidative stress [100]. Many other nanoparticles targeting oxidative stress as a theranostic strategy for CVD have also been actively developed and evaluated [101].

#### 4.3.2. Gene Therapy

Gene therapy is a type a therapeutic approach that seeks to modify the expression of certain genes in order to alter certain biological properties, which has gained significant amount of interest in recent years [102]. Whether by replacing the disease-causing gene with a healthy gene, or inactivating the disease-causing gene, or introducing a new or modified gene to treat the existing disease, gene therapy requires precise targeting [102]. With the help of nanoparticles for targeted delivery, we are getting closer to the realization of gene therapy being used in the clinical setting.

Cationic PEG-PLA nanoparticles is used as a major delivery system to deliver small interference RNA (siRNA) [11]. PEG-PLA nanoparticles encapsulating siRNA can enter the cells to perform gene-specific knockdown [103]. However, challenges still remain for these biodegradable nanoparticle-assisted anti-cancer therapies to come to realization in the clinics. CALAA-01, an anti-solid tumor nanoparticle containing siRNA, showed great potential in its phase I clinical trial (NCT00689065), was terminated after phase Ib as two of the five patients enrolled had experienced dose-limiting toxicities [104,105]. CRLX101, another anti-tumor targeted nanoparticle for various cancers, continues to show promising results and its clinical trial (NCT02769962) is still actively recruiting patients [106]. In 2019, the first ever siRNA nanodrug for hereditary amyloidosis, Onpattro, was approved by the FDA [107]. Onpattro encapsulates the therapeutic siRNA moiety into a lipid nanoparticle, and delivers it directly to the liver to prevent the body from producing the disease-causing amyloid proteins [107].

Similarly, research on stem cell therapy utilizing nanoparticles is also on the rise. Because of the small size and target specificity of the nanoparticles, scientists are aiming to treat some neurological diseases using this strategy [108].

#### 4.3.3. Oral Drug Delivery

Another active area of research using biodegradable nanomaterials is to make it possible for certain drugs that are normally either poorly or erratically absorbed in the digestive system to be administered orally. This approach can ease the administration process of many biologics, proteins and peptides. For example, insulin is a peptide that is digested in the stomach [109]. Almost a century after the discovery of insulin, it can still only be administered via subcutaneous injection, adding not only physical discomfort and infection risks, but also psychological burdens to the diabetic patients. Research has been underway to make oral administration of insulin possible in order improve the quality of life of the diabetic patients [109]. A specific formulation of 1.6% zinc insulin in PLGA was developed in 2010 in an effort to realize the oral administration of insulin [109]. Although this PLGA nanoparticle encapsulating insulin only showed 11.4% of the efficacy of zinc insulin via intraperitoneal delivery, it still shed light to a possible future of oral administration of insulin [109]. More recently, combinations of different biodegradable nanomaterials, including chitosan, have also been applied in the development of oral delivery system of insulin [110,111]. Some phase I/II clinical trials are also underway [110].

### 4.4. Implantable Device with Biodegradable Materials

Continuous efforts have been putting forward to improve the outcomes of implantable devices using biodegradable materials. Biodegradable nanoparticles not only are employed in the nanodrug delivery systems but can also be incorporated in the implantable devices such as orthopedic fixation devices (including fracture-fixation pins and plates, interference screws, suture anchors, craniomaxillofacial fixation devices and tacks for meniscal repair), and biodegradable stents for percutaneous coronary intervention [112,113]. A number of these devices have already been approved and are available in the market [112]. The biodegradable nanoparticles enable the implanted devices to gradually degrade while the host tissues undergo constructive remodeling, eventually replacing the implant [112].

Significant progress has been made especially in interventional cardiology [114]. New drug-eluting stents have been developed to not only minimize neointimal hyperplasia and reduce restenosis after revascularization, but also minimize stent thrombosis, a problem that was observed at higher frequency with the first generation stents [114]. Recently, Lih et al. developed a new approach to prevent acid-induced inflammatory responses associated with biodegradable PLGA, by neutralizing the acidic environment using oligo(lactide)-grafted magnesium hydroxide (Mg(OH)_2_) nanoparticles [115]. They demonstrated in porcine models that incorporating the modified Mg(OH)_2_ nanoparticles within degradable coatings on drug-eluting arterial stents could efficiently attenuate the inflammatory response and in-stent intimal thickening [115]. Their results suggested that modifications of biodegradable nanoparticles could be useful to broaden the applicability and improve clinical success of biodegradable devices used in various biomedical fields. Biodegradable stents were invented with the intention to replace bare metal stents due to the high risk of in-stent restenosis using the metal materials [116]. However, great challenge still persists to achieve the right balance of the polymer, drug and degradation rate in order to avoid acute or chronic recoil and maintain vessel patency after stent implantation [116].

Taken together, biodegradable nanomaterials have shown many advantages in various biomedical applications. Here in Table 2 we highlight some of the above mentioned advantages to illustrate the ability of these biodegradable nanomaterials in meeting different clinical needs.

## 5. Current Status of Biodegradable Nanomaterials and Challenges Ahead

Nanotoxicity, defined as toxicity induced by nanomaterials, is still an important discipline of research as the human body is being increasingly exposed to foreign materials at a nanoscale with the development of nanomedicine either intentionally or unintentionally [95]. Even with biodegradable nanomaterials, safety assessment remains as one of the top priorities in the application of nanomedicine. The toxicity of nanomaterials has been largely decreased with the application of biodegradable materials, even when sometimes the toxicity of the payload is unavoidable for some treatments [117]. However, it needs to be noted that not all biodegradable materials are deemed safe for application in humans. Even with biodegradability, some nanoparticles may still have undesired effects on the blood coagulation system due to their physiochemical properties such as size, charge and hydrophobicity [118]. The PLGA and PLA for clinical applications are manufactured under current good manufacturing practice protocols regulation by the FDA to ensure efficacy, safety, and stability for pharmaceuticals [11]. However, poly-alkyl-cyanoacrylate (PAC), for instance, can be degraded by esterases in the body but the degradation process produces toxic components [8,119]. Given the relatively short history of nanomedicine, the long-term effects of newly developed nanoparticles still need to be carefully evaluated.

The bio-distribution and pharmacokinetics of nanoparticles are largely dependent on the size, shape and the surface charge of the nanoparticles applied [117,120]. The early challenge of premature denaturation and undesired biodistribution of nanoparticles due to non-specific protein adsorption forming a protein corona around the material when being exposed to the biological environment has been solved by PEGylation [121]. However, it is still crucial to control the degradation rate of the nanomaterials and the payload’s releasing profile since these biodegradable nanomaterials will eventually be degraded. The choice of nanomaterial also influences the outcome of certain nanotherapeutics. For example, PCL has a much lower encapsulation efficiency for taxol, an anti-cancer drug, compared to PLGA (20% vs. 100%) [122]. However, PCL nanoparticles have better therapeutic efficiency and stability than PLGA nanoparticles [122].

There is continuous advancement of the nanotechnologies using biodegradable materials in nanomedicine. Literature search using the PubMed database revealed that about 60% of all the studies on biodegradable nanomaterials in nanomedicine were from the past 5 years. However, the translation of different novel biodegradable nanoparticle designs into clinical settings remains a huge challenge. Current nanoparticle production methods are still constrained by several limitations, including the relatively high cost of particle production with difficulty in synthesizing particles that are homogeneous in shape and size; the low drug encapsulation efficiency; the difficulty in large scale production and sterilization; and the lack of reliable method for releasing profile measurement with the potential problem of high initial burst release or incomplete drug release [11]. Furthermore, there is still huge unknown regarding the correlation between nanoparticles’ properties and their in vivo behaviors, their long-term stability, and how would some residual materials used for nanoparticle modification affect the human body [11]. As many nanoparticles have their unique structures and compositions which lead to their unique properties, there is also unmet need to develop standardized test protocols as well as reference particles for validation [11].

All these challenges call for the need to collect comprehensive information of biodegradable nanomaterials, drugs, as well as human data for the optimal modification and application of the nanoparticles and drugs. Even with this systemic review, we are still at risk of falling in the underreporting bias category as the studies were screened and selected manually. Using machine learning and artificial intelligence (AI), the properties of different nanomaterials and different combinations can be screened and the behavior of combinatorial nano-bio interface can be predicted [123,124]. The screening and model development might also lead to new discoveries of potential biodegradable nanomaterials and nanoparticle designs. Therefore, as for the future of the development of biodegradable nanomaterials in nanomedicine, machine learning and AI will be a great asset to the realization of efficient bench to bedside translation as well as personalized nanomedicine. Given the fast development of this field in the past few years, it is likely that in the near future, more newly developed biodegradable nanoparticles, especially multimodal nanoparticles, will be evaluated in clinical trials for their potential translational use.

## 6. Conclusions

In this review, we discussed different types of biodegradable nanomaterials and their applications in the biomedical field. These materials have demonstrated superiority compared to non-degradable counterparts and hold great translational potential in various clinical settings. There is still great challenge in developing nanomedicine and more biodegradable nanomaterials remain to be explored and validated for their potential clinical use.

## Figures and Tables

**Table 1:** Advantages and limitations of some biodegradable nanoparticles.

Biodegradable Nanoparticles		Advantages	Limitations
General		Biocompatibility Low immunogenicity Slow and controlled release of the cargo Targeted delivery Enhanced therapeutic effects Decreased side-effects and cytotoxicity Modifiable size and surface to improve drug release profile and targeting efficiency	Instable if not modified High cost of particle production Difficult to synthesize particles that are homogeneous in shape and size Relatively low drug encapsulation efficiency Difficulty in large scale production and sterilization
Polymer-based	PLA micelles	Hydrophobicity useful for carrying low soluble drugs Easily modifiable physical and chemical properties to obtain desirable pharmacokinetic and biodegradable properties	Non-specific uptake by the reticuloendothelial systems Low drug loading capacity Low encapsulation efficiency Particle size-dependent immunotoxicity Easily affected degradation rate
	PLGA micelles	Wide range of erosion times Modifiable mechanical properties Degradation rate can be changed by adjusting the ratio of PLA:PGA and their molecular weights	The acidic nature of PLGA monomers are not suitable for certain drugs and bioactive molecules Difficult to achieve optimal drug release profile non-linear, dose-dependent, and easily altered biodistribution and pharmacokinetics
	PCL nanoparticles	Degradation does not produce acidic byproducts Slow degradation rate, ideal for long-term implantation device Versatile mechanical properties	Hydrophobicity that limits its production
	Chitosan nanoparticles	Good absorbability, permeability, and moisture retention Easily degradable Low toxicity	Very sensitive to environmental temperature Degradation rate affected by environmental pH Poor long-term stability
	Dendrimers	High degree of branching and polyvalency, with very high surface-to-volume ratio, enabling high drug carrying efficiency Capable of carrying drugs with poor solubility High water solubility Useful as MRI imaging agent	Cytotoxicity may occur during the interactions with cell membrane depending on the charge and modifications made on the surface of the dendrimer
Lipid-based	Liposomes	Self-assembly, enabling easy drug loading High loading efficiency Protect encapsulated drugs from early inactivation, degradation and dilution in the circulation Can be formulated into different forms for various routes of administration Easily functionalized with surface modification	Fast clearance rate Low stability Complex production method

**Table 2:** Biomedical applications of biodegradable nanomaterials.

Purpose	Application	Advantages of Biodegradable Nanomaterials	References
Imaging	MRI	Reduced contrast load Stronger signals Targeted imaging	[66,67,68,69,70,71,72,73,74,75,76]
	photoacoustic imaging		
Theranostics	photoacoustic imaging and photothermal therapy	Safely facilitate real-time therapeutic efficacy towards individualized treatment strategies	[78,79,80,81,82,83,84,85,86,87]
Targeted Delivery(carried by liposomes, polymeric nanoparticles, dendrimers, or micelles)	Drug delivery	Encapsulation of hydrophobic molecules Reduced premature degradation Improved drug uptake Sustained drug concentrations within the therapeutic window Reduced side effects	[88,89,90,91,92,93,94,95,96,97,98,99,100,101]
	Gene therapy	Protect DNA from enzymatic degradation Reduced rejection from host immune system	[102,103,104,105,106,107]
	Antigen delivery	Controlled release of antigen/ligand Protect the antigen load	[112]
Implants	Stents	Reduced vessel occlusion, restenosis, or late stent thrombosis Improved lesion imaging No need for a secondary surgical removal	[112]
	Mesh	No need for a secondary procedure to remove the mesh	[112]
	Suture	Low immunogenicity and toxicity Excellent biocompatibility Predictable biodegradation rates Good mechanical properties	[112]
